# Isolation, identification, and pathogenicity of *Steinernema carpocapsae* and its bacterial symbiont in Cauca-Colombia

**DOI:** 10.21307/jofnem-2020-089

**Published:** 2020-08-31

**Authors:** Esteban Neira-Monsalve, Natalia Carolina Wilches-Ramírez, Wilson Terán, María del Pilar Márquez, Ana Teresa Mosquera-Espinosa, Adriana Sáenz-Aponte

**Affiliations:** 1 Biología de Plantas y Sistemas Productivos, Departamento de Biología, Pontificia Universidad Javeriana, Bogotá, Colombia; 2 Departamento de Ciencias Naturales y Matemáticas, Pontificia Universidad Javeriana, Cali, Colombia

**Keywords:** Biological control, Colombia, *Epitrix cucumeris*, *Pandeleteius cinereus*, *Steinernema carpocapsae*, *Xenorhabus nematophila*

## Abstract

In Colombia, identification of entomopathogenic nematodes (EPN’s) native species is of great importance for pest management programs. The aim of this study was to isolate and identify EPNs and their bacterial symbiont in the department of Cauca-Colombia and then evaluate the susceptibility of two Hass avocado (*Persea americana*) pests to the EPNs isolated. EPNs were isolated from soil samples by the insect baiting technique. Their bacterial symbiont was isolated from hemolymph of infected *Galleria mellonella* larvae. Both organisms were molecularly identified. Morphological, and biochemical characterization was done for the bacteria. Susceptibility of *Epitrix cucumeris* and *Pandeleteius cinereus* adults was evaluated by individually exposing adults to 50 infective juveniles. EPNs were allegedly detected at two sampled sites (natural forest and coffee cultivation) in 5.8% of the samples analyzed. However, only natural forest EPN’s could be isolated and multiplied. The isolate was identified as *Steinernema carpocapsae* BPS and its bacterial symbiont as *Xenorhabus nematophila* BPS. Adults of both pests were susceptible to *S. carpocapsae* indicating this EPN potential for its management. The results of this study constitute the first record of *S. carpocapsae* in Colombia and the susceptibility of *P. cinereus* to this EPN.

Entomopathogenic nematodes (EPNs) of the Steinernematidae and Heterorhabditidae families are widely used as biological control agents that represent a promising alternative to replace pesticides (Labaude and Griffin, 2018), because of their ability to parasitize insects, being able to identify, locate, and infect a host and to kill it within 48 hr, as well as they are safe to vertebrates, plants, and other non-target organisms. Moreover, they can be applied by means of standard spraying equipment (Poinar, 1972; Hazir et al., 2003; Van Zyl and Malan, 2014), and can be propagated in mass either in vivo using host insects such as *Galleria mellonella* (Lepidoptera: Pyralidae) or in vitro in bioreactors using artificial culture media (Shapiro-Ilan et al., 2014).

EPNs life cycle has six states: egg, four juvenile stages, and adults: males, females, or hermaphrodites (Shapiro-Ilan et al., 2014). EPNs of the Steinernematidae family are characterized by symbiotic associations with bacteria of the genus *Xenorhabdus*, while EPNs of the Heterorhabditidae family have symbiotic associations with bacteria of the genus *Photorhabdus*. In these relationships, the nematode acts as a vector allowing the bacteria to reach and enter the host insect where they produce metabolites that kill it within 24 to 48 hr, degrade it and transform it into an optimal environment for the development of EPNs. Almost every stage of EPNs development takes place inside the insect. The exception is the infective juvenile (IJ) which is a modification of the J3 stage that does not feed or develop, responsible for actively mobilizing in the soil in search of potential hosts and for carrying the bacterial symbionts in the intestinal lumen (*Heterorhabditis* sp.), or in a specialized structure called the receptacle; a modification of the two most anterior intestinal cells (*Steinernema* sp.) (Griffin et al., 2005; Stock, 2015).

Because various environmental conditions can affect survival, reproductive potential, and virulence of EPNs, the use of native species for pest control is of great importance since they are better adapted to local environmental conditions than are foreign species (Campos-Herrera and Gutiérrez, 2009). Nevertheless, in a number of countries information on EPNs and their bacterial symbionts is scarce (De Brida et al., 2017). Colombia’s records of native EPNs, a tropical country with a large agricultural population and sector, do include *Steinernema websteri*, *S. kraussei*, *S. colombiense*, and *Heterorhabditis bacteriophora* (López-Núñez et al., 2007, 2008; Melo et al., 2009). The municipality of Toribío, in the Department of Cauca, Colombia, is a place where EPNs had never been registered before. It is characterized by small-scale family and community agriculture with varied subsistence and pasture crops. The aim of this study was to isolate and identify EPNs and their bacterial symbiont from crops and natural habitats at this municipality for later in vitro evaluation of susceptibility of two insect pests of economic interest for Hass avocado crops (*Persea americana*), the potato flea beetle (*Epitrix cucumeris* [Coleoptera: Chrysomelidae]), and a broad-nosed weevil (*Pandeleteius cinereus* [Coleoptera: Curculionidae]), to the EPNs isolated and the reference isolate *Steinernema carpocapsae* FA2015.

## Materials and methods

### Isolation and identification of entomopathogenic nematodes

#### Soil sampling

Soil samples were taken in the Toribío and Tacueyó reserves of the municipality of Toribío, in the Department of Cauca, Colombia, where EPNs have never been applied. These rural areas are located between 1,757 and 2,963 meters above sea level (m.a.s.l) where temperatures range between 13.9° and 20°C. Following the sampling method developed by Varón de Agudelo and Castillo (2001), 10 sampling sites in eight types of vegetation cover were selected: natural grassland (NG), pasture bordering annual crops (PBAC), natural forest (NF), first-growth American bamboo (*Guadua* sp.) (FGAB), coffee cultivation (*Coffea arabica*) (CC), horticultural cultivation (HC), strawberry cultivation (*Fragaria* sp.) (SC), and combined lulo (*Solanum quitoense*) and papaya cultivation (*Carica* sp.) (CLPC). At each site, two composite soil samples (10 subsamples, ~1 kg) were taken at depths between 20 and 30 cm, after removing soil cover to avoid cross contamination. Each independent composite sample was taken in an area measuring approximately 16 m^2^ with a zigzag distribution of 10 subsampling points. Samples were packed in plastic bags, labeled, and stored in a cooler (~15°C) (Orozco et al., 2014) in dark conditions for less than 24 hr until they arrived at the Biological Control laboratory of Pontificia Universidad Javeriana in Bogotá, Colombia to be immediately processed.

#### Isolation of EPNs

Composite soil samples taken from each site were mixed and stored at room temperature for 24 hr prior to testing for IJs using the insect baiting technique (Bedding and Akhurst, 1975). For this, six samples of 150 g of mixed soil were deposited in 200 cm^3^ plastic boxes. Then, five *G. mellonella* larvae and five *Tenebrio molitor* (Colepotera: Tenebrionidae) larvae were added per box at the soil surface. The boxes were labeled, inverted, and kept in darkness at 20°C. Four days later, the boxes were evaluated for dead larvae killed by EPNs. Sagging bodies and color changes were noted. Subsequently, dead larvae were placed individually in White traps (White, 1927) to obtain IJs. The IJs recovered were then multiplicated in *G. mellonella* larvae and maintained for later identification. To corroborate whether EPNs were absent or present in soil samples, samples were processed twice by repeating the process described above.

The IJs isolated were stored in polyurethane foams at 10°C in the Biological Control laboratory and deposited in the Entomology collection of the Javeriana Museum of Natural History of the Pontificia Universidad Javeriana in Bogotá, Colombia.

Abundance of EPNs at sampled sites (positive sites for EPNs/total sites) and the recovery frequency of EPNs (positive samples for EPNs/total samples analyzed) were determined according to the type of vegetation cover (Liu and Berry, 1995) and expressed as a percentage.

### Molecular identification

#### DNA extraction

Genomic DNA was extracted individually from females following the protocol described by Çimen et al. (2016). After lysis of the nematodes, protein content was separated using NaCl at a final concentration of 1.7 M and centrifuging at 3,000 g for 15 min at room temperature. Finally, the supernatant was transferred to another tube for alcohol precipitation of DNA.

#### Amplification and sequencing of taxonomic markers

Sequences of the following three taxonomic markers commonly used for nematodes were amplified by PCR and used for molecular identification: a fragment of the 18S rRNA sequence using primers SSU18A-4F: 5′-GCTTGTCTCAAAGATTAAGCCATGCATG-3′ and SSU26Rplus4: 5′-AAGACATTCTTGGCAAATGCTTTCG-3′ (Morise et al., 2012); a fragment that contained the sequences ITS1, 5.8S, and ITS2 using primers 18S: 5′-TTGATTACGTCCCTGCCCTTT-3′ and 26S: 5′-TTTCACTCGCCGTTACTAAGG-3′ (Vrain et al., 1992); and a rRNA 28S fragment that contained the D2/D3 expansion sequence using D2F: 5′-CCTTAGTAACGGCGAGTGAAA-3′ (Nguyen et al., 2006) and 536: 5′-CAGCTATCCTGAGGAAAC-3′ as primers (Stock et al., 2001).

For fragment amplification, 50 µl of PCR mixture was prepared using 1X NH_4_ Buffer (Bioline, England), 3 mM MgCl_2_, 0.2 mM dNTPs, and 0.5 µg/µl Bovine Serum Albumin (New England Biolabs, United States), 0.5 µM forward primer, 0.5 µM reverse primer, Taq DNA polymerase 2U (Bioline, England), and 100 to 200 ng of template DNA (Hominick et al., 1997; Stock et al., 2001).

The PCR protocol for the 18S fragment consisted of one initial denaturation cycle at 94°C for 2 min followed by 35 cycles at 94°C for 10 sec, 55°C for 30 sec, 68°C for 1 min (Morise et al., 2012), and a final extension at 68°C for 7 min. For the ITS and D2/D3 fragments, the PCR protocol consisted of one cycle of initial denaturation at 94°C for 7 min followed by 35 cycles at 94°C for 1 min, 1 min at the annealing temperature (ITS: 50°C and D2/D3: 55°C), 72°C for 1 min and a final extension at 72°C for 7 min (Çimen et al., 2016).

Amplification of all PCR products and their respective negative amplification controls were verified by 1% (w/v) agarose gel electrophoresis in 1X TBE buffer stained with 0.5 X Hydragreen (ACTGene, United States). PCR products were purified for sequencing with Wizard SV Gel and PCR clean-up system (Promega, United States). Three purified amplicons obtained from three independent DNA extractions were sequenced for each molecular marker. Sequencing of each amplicon was performed in both directions.

#### Processing of the obtained sequences

The six sequences obtained for each marker were visualized, aligned, and edited manually using MEGA X software (Kumar et al., 2018) in order to obtain a consensus sequence. Its identity was initially verified by means of the Basic Local Alignment Search Tool (BLAST) (Altschul et al., 1990) on the basis of the non-redundant (nr) database of the NCBI. Consensus sequences of all markers were deposited in GenBank (NCBI) under the accession numbers listed in Table 1.

**Table 1.** Accession numbers of sequences used for phylogenetic analysis of the EPN isolated and its bacterial symbiont.

	Gen				
Species	18S		ITS	28S	
*Steinernema carpocapsae* BPS	MK558002 ■		MK558041 ■	MK558056 ■	
*Steinernema carpocapsae*	KJ636405		AF121049	KJ950293	
*Steinernema arenarium*	KJ636393		KU194614	KU194619	
*Steinernema feltiae*	KJ636413		AF121050	JF920963	
*Steinernema glaseri*	KU180674		AF122015	GU177831	
*Steinernema bicornutum*	KT878311		AF121048	GU569045	
*Steinernema poinari*	KT878314		KF241753	KF241750	
*Steinernema akhursti*	KT878310		DQ375757	KF289902	
*Steinernema karii*	AJ417021		AY230173	AF331902	
*Steinernema beitlechemi*	KT878316		KT373856	KT580949	
*Steinernema kushidai*	LC157426		GQ497741	AF331897	
*Steinernema affine*	FJ040425		AY230159	AF331899	
*Steinernema websteri*	–		FJ381666	AY841762	
*Steinernema scarabaei*	–		FJ263673	AY172023	
*Steinernema khuongi*	–		GU174002	GU177835	
*Steinernema diaprepesi*	–		AF122021	GU177828	
*Steinernema hermaphroditum*	–		MF663703	MF693228	
*Steinernema rarum*	–		DQ221115	AY253296	
*Steinernema intermedium*	–		AF122016	AF331909	
*Steinernema unicornum*	–		GQ497167	GU191462	
*Steinernema scapterisci*	–		AF122020	AF331898	
*Steinernema neocurtillae*	–		AF122018	FJ263674	
*Steinernema monticolum*	–		AF122017	AF331895	
*Steinernema nguyeni*	–		KP325084	KR815816	
*Steinernema sacchari*	–		KC633095	KC633096	
*Steinernema phyllophagae*	–		FJ410327	FJ666054	
*Steinernema pakistanense*	–		MF289981	JX068823	
*Steinernema australe*	–		FJ235125	FJ235126	
*Steinernema brazilense*	–		FJ410325	FJ410326	
*Steinernema riobrave*	–		AY230182	GU177834	
*Steinernema cubanum*	–		AY230166	AF331889	
*Steinernema ceratophorum*	–		AY230165	AF331888	
*Steinernema texanum*	–		EF152568	EF152569	
*Steinernema cholashanense*	–		MF039642	EF520284	
*Steinernema apuliae*	–		HQ416968	KU194621	
*Steinernema khoisanae*	–		DQ314287	DQ314289	
*Steinernema aciari*	–		AY787660	GU395637	
*Steinernema citrae*	–		FJ235074	MF540678	
*Steinernema silvaticum*	–		MG543848	MG547579	
*Steinernema surkhetense*	–		MF919614	KU187262	
*Steinernema litorale*	–		JF892546	JQ795723	
*Steinernema anatoliense*	–		EU200356	AY841761	
*Caenorhabditis elegans*	X03680		X03680	X03680	
*Panagrellus redivivus*	–		–	AF331910	
	16S	*dnaN*	*recA*	*gltX*	*rplB*
*Xenorhabdus nematophila* BPS	MK558196 ■	MK570079 ■	MK570081 ■	MK570080 ■	MK570082 ■
*Xenorhabdus nematophila* ATCC19061	D78009	NC_014228	AF127333	NC_014228	CBJ88402
*Xenorhabdus beddingii*	AY278675	FJ831460	FJ823415	FJ840506	FJ808999
*Xenorhabdus bovienii*	X82252	FJ831466	FJ823426	FJ840514	FJ809005
*Xenorhabdus japonica*	DQ202310	FJ831453	FJ823400	FJ840503	FJ808989
*Xenorhabdus poinarii*	D78010	FJ831454	FJ823409	FJ840499	FJ808995
*Xenorhabdus szentirmaii*	AJ810295	FJ831458	FJ823416	FJ840508	FJ809002
*Xenorhabdus ehlersii*	AJ810294	FJ831448	FJ823398	FJ840495	FJ808992
*Xenorhabdus innexi*	AJ810292	FJ831476	FJ823424	FJ840522	FJ809018
*Xenorhabdus budapestensis*	AJ810293	FJ831474	FJ823418	FJ840518	FJ809017
*Xenorhabdus indica*	AM040494	FJ831470	FJ823421	FJ840520	FJ809013
*Xenorhabdus cabanillasii*	AY521244	FJ831472	FJ823422	FJ840521	FJ809015
*Xenorhabdus doucetiae*	DQ211709	FJ831450	FJ823402	FJ840497	FJ809001
*Xenorhabdus griffiniae*	DQ211710	FJ831449	FJ823399	FJ840496	FJ808991
*Xenorhabdus hominickii*	DQ211719	FJ831461	FJ823410	FJ840510	FJ809010
*Xenorhabdus koppenhoeferi*	DQ205450	FJ831457	FJ823413	FJ840504	FJ809004
*Xenorhabdus kozodoii*	DQ211716	FJ831446	FJ823404	FJ840493	FJ808994
*Xenorhabdus mauleonii*	DQ211715	FJ831464	FJ823417	FJ840507	FJ809003
*Xenorhabdus miraniensis*	DQ211713	FJ831459	FJ823414	FJ840505	FJ808990
*Xenorhabdus romanii*	DQ211717	FJ831451	FJ823403	FJ840498	FJ809000
*Xenorhabdus stockiae*	DQ202309	FJ831477	FJ823425	FJ840524	FJ809020
*Xenorhabdus vietnamensis*	DQ205447	FJ831452	FJ823401	FJ840502	FJ808998
*Xenorhabdus ishibashii*	GQ149086	JQ348908	JQ348906	JQ348909	PHM62307
*Xenorhabdus magdalenensis*	HQ877464	JF798399	JF798401	JF798400	-
*Xenorhabdus khoisanae*	HQ142625	AB685733	AB685736	AB685734	KMJ46808
*Xenorhabdus thuongxuanensis*	KX602193	KX602195	KX602194	KX602196	OKP02162
*Xenorhabdus eapokensis*	KX602187	KX602189	KX602188	KX602190	OKP00696
*Morganella morganii* subsp. *morganii*	AJ301681	–	–	–	–
*Photorhabdus luminescens* subsp*. laumondii*	–	FJ831497	KT963835	KT963845	FJ817457
*Proteus mirabilis*	–	NC_010554	X14870	CAR43779	CAR46384

**Note:** ■ sequences obtained in this study.

#### Phylogenetic analysis

The consensus sequences of all markers were subsequently aligned with sequences of various species of EPNs deposited in GenBank (Table 1) using MUSCLE (Edgar, 2004) in MEGA X software (Kumar et al., 2018). The alignment obtained was edited so that all sequences used for analysis were of the same length and same genetic region.

Then, phylogenetic trees were independently constructed for each marker. The Tamura–Nei model of maximum likelihood estimation (Tamura and Nei, 1993) with gamma distribution and 1,000 bootstraps was used for the 18S fragment, and the General Time Reversible model of maximum likelihood estimation (Nei and Kumar, 2000) with gamma distribution and 1,000 bootstraps was used for the ITS fragment. Unweighted Pair Group Method with Arithmetic Mean (UPGMA) (Sneath and Sokal, 1973) with gamma distribution and 1,000 bootstraps was used for the D2/D3 fragment of rRNA 28S.

Because of the small number of sequences available for the 18S marker in GenBank, the additional phylogenetic analysis of concatenated sequences was made only with ITS (601 nts) and D2/D3 (754 nts) fragments. This phylogenetic tree was constructed using UPGMA (Sneath and Sokal, 1973) (Tamura–Nei model of maximum likelihood estimation (Tamura and Nei, 1993) with gamma distribution and 1,000 bootstraps).

### Susceptibility of *Epitrix cucumeris* and *Pandeleteius cinereus* adults to EPNs

The susceptibility of *E. cucumeris* and *P. cinereus* adults (Hass avocado [*P. americana*] pests) to the EPNs isolated, and *S. carpocapsae* FA2015 as reference (commercially obtained), was evaluated and compared in vitro. Adults of both insect species were collected from a *P. americana* crop. In vitro bioassays were performed in multicell culture plates containing sterile Whatman No. 1 filter paper (GE Healthcare, United States). In total, 10 adults of each insect species were exposed individually to 0 IJs (sterile distilled water) and 50 IJs of each EPN. Viability of IJs was checked during the count before adding them to the plates. Plates were incubated at 26°C for 8 days, and adult survival was recorded every 24 hr.

To corroborate whether mortality was due to EPNs and check for EPNs reproduction inside the insects, adults were dissected in Ringer solution (9 g/liter NaCl, 0.4 g/liter KCl, 0.4 g/liter CaCl_2_, 0.2 g/liter NaHCO_3_) (Merck, United States). Each bioassay was repeated three times over time.

### Statistical analysis

Survival analysis of insects exposed to the EPNs was performed using Graphpad Prism 6.01 software (GraphPad Software, United States) using the Kaplan–Meier method (Kaplan and Meier, 1958). To identify any significant differences between treatments, we used analysis of variance (ANOVA) testing and multiple comparisons with the Tukey test using the area under the curve.

### Isolation and identification of bacterial symbiont

#### Isolation

Bacterial symbiont was isolated from hemolymph of *G. mellonella* larvae infected with the EPNs recovered, following the procedure described by Kazimierczak et al. (2017). After isolation, bacterial symbionts were stored at −80°C in LB Broth (Scharlab, Spain) with 20% (v/v) glycerol (Merck, United States) and deposited in the Microorganisms Collection of Pontificia Universidad Javeriana – CMPUJ.

#### Confirmation of bacterial symbiont’s isolate

The correspondence of bacterial isolates to what is expected for EPNs bacterial symbionts of the *Xenorhabdus* or *Photorhabdus* genus (Akhurst and Boemare, 2015; Boemare and Akhurst, 2015) was initially confirmed through microscopic and macroscopic morphological characteristics by Gram staining and, on NBTA (13 g/liter nutrient broth, 0.025 g/liter bromothymol blue, 0.04 g/liter chloride 2, 3,5-triphenyltetrazolium, and 15 g/liter agar), MacConkey (Scharlab, Spain) and blood agar (Becton, Dickinson and Company, United States). Samples were incubated at 26°C for 48 hr. To corroborate symptoms of *G. mellonella* larvae infected with the bacterial isolates, a colony was removed from NBTA medium after 48 hr growth at 26°C, and resuspended in saline solution (0.85% (w/v) NaCl [Merck, United States]). The suspension was serially diluted to 10^−4^. Then, 10 µl of the last dilution was injected into five larvae of *G. mellonella* with a syringe (Hamilton Company, United States). The surface of the larvae was disinfected with 1% NaClO (BNS S.A, Colombia) for 1 min, followed by three washes with sterile distilled water. As a negative control, 10 µl of sterile saline solution was injected into five larvae of *G. mellonella*.

Following injection, larvae were incubated at 26°C for 48 hr, after then, their coloration, mortality, and consistency were all checked.

#### Biochemical characterization

To identify metabolic characteristics of the bacterial isolate, a colony of the bacteria was exposed to a 3% hydrogen peroxide solution (Sigma-Aldrich, United States) using sterile wooden sticks. Subsequently, a colony was taken from nutrient agar (Scharlab, Spain) after 48 hr growth at 26°C and then resuspended in 0.85% API NaCl medium (BioMérieux, France). The API 20E fast identification system (BioMérieux, France) was then used according to the manufacturer’s instructions and incubated at 26°C for 48 hr. After incubation, results were read and interpreted following the manufacturer’s instructions.

#### Molecular identification

Bacterial genomic DNA was extracted following the CTAB-based DNA extraction protocol described by Feil et al. (2004). From the extracted DNA, a Multilocus Sequence Typing (MLST) analysis was performed with the partial sequences of five genes used as taxonomic markers. The following five sequences and primers were used: 16S rRNA using 16SP1 5′-GAAGAGTTTGATCATGGCTC-3′ and 16SP2 5′-AAGGAGGTGATCCAGCCGCA-3′ (Tailliez et al., 2006); *dnaN* (DNA polymerase III beta chain) using dnaN1 5′-GAAATTYATCATTGAACGWG-3′ and dnaN2 5′-CGCATWGGCATMACRAC-3′; *recA* (DNA recombinase) using recA1 5′-GCTATTGATGAAAATAAACA-3′ and recA2 5′-RATTTTRTCWCCRTTRTAGCT-3′; *gltX* (glutamyl-tRNA synthetase) using gltX1 5′-GCACCAAGTCCTACTGGCTA-3′ and gltX2 5′- GGCATRCCSACTTTACCCATA-3′; and *rplB* (50S subunit of ribosomal protein L2) using rplB1 5′-GGCAATTGTTAAATGTAAACC-3′ and rpblBXeno2 5′-GCGGCGTACGATGTATTGAT-3′ (Tailliez et al., 2010).

For molecular markers amplification, 25 µl of PCR mixture was prepared using 1X NH_4_ Buffer, 3 mM MgCl_2_, 0.2 mM dNTPs (Bioline, England), 0.5 µg/µl Bovine Serum Albumin (New England Biolabs, United States), forward primer and reverse primer for each 0.5 µM of taxonomic marker, Taq DNApolimerase 2U (Bioline, England) and 20 to 100 ng of bacterial DNA (Hominick et al., 1997; Tailliez et al., 2006).

The PCR protocol consisted of an initial denaturation at 94°C for 5 min, 30 cycles at 94°C for 30 sec, 30 sec at the annealing temperature of each pair of primers (16S: 64.9°C, *dnaN*: 45°C, *recA*: 45°C, *gltx*: 64.9°C and *rplB*: 58.3°C), extension at 72°C for 1 min, and a final extension at 72°C for 7 min. The specific amplification of all PCR products was verified by electrophoresis in 1% (w/v) agarose gel in 1X TBE buffer stained with 0.5 X Hydragreen (ACTGene, United States).

PCR products were purified for sequencing with Wizard SV Gel and PCR clean-up system (Promega, United States). Three independent amplicons were sequenced in both directions for each molecular marker.

#### Sequence processing

The six sequences obtained for each marker were visualized, aligned, and edited manually using the MUSCLE tool (Edgar, 2004) in the MEGA X software (Kumar et al., 2018) in order to obtain the respective consensus sequence. The consensus sequence for 16S marker was initially identified by means of EzBioCloud (Yoon et al., 2017), while BLAST (Altschul et al., 1990) was used for initial identification of other markers. The consensus sequences of all markers were deposited in GenBank (NCBI) under the accession numbers registered in Table 1.

#### Phylogenetic analysis

The consensus sequences of each marker were aligned with sequences of species deposited in GenBank (NCBI) using MUSCLE (Edgar, 2004) in MEGA X software (Kumar et al., 2018). The alignment obtained was edited so that all the sequences used in the analysis were of the same length and genetic region, and phylogenetic trees were constructed independently for each marker. Maximum likelihood estimation (Tamura–Nei model [Tamura and Nei, 1993] with gamma distribution, invariant sites (G + I), and 100 bootstraps) was used for the 16S fragment; maximum likelihood estimation (Kimura 2 model [Kimura, 1980] with gamma distribution, invariant sites (G + I), and 100 bootstraps) was used for the *dnaN* and *rplB* fragments; and maximum likelihood estimation (Tamura 3 model [Tamura, 1992] with gamma distribution, invariant sites (G + I), and 100 bootstraps) was used for the *gltX* and *recA* fragments.

In addition, the *dnaN* (815 nts), *gltX* (654 nts), *recA* (592 nts), and *rplB* (678 nts) markers were manually concatenated for construction of the phylogenetic tree using maximum likelihood estimation (General Time Reversible model (Nei and Kumar, 2000) with gamma distribution, invariant sites [G + I], and 100 bootstraps). The accession numbers of all the sequences used are found in Table 1.

## Results

### Isolation and identification of entomopathogenic nematodes

Only 14 out of 240 soil samples (5.8% recovery frequency) were found to contain EPNs. They were all from two of the 10 sites sampled (20% abundance) (site 1: CC and site 2: NF) (Table 2). However, it was only possible to multiply and maintain the isolate that was found in the soils of site 2. Only dead IJs were found when larvae with symptons of NEPs from site 1 were dissected.

**Table 2.** Isolation of EPNs according to types of vegetation cover at sampling sites.

Vegetation cover	Location	Altitude (m.a.s.l)	Soil temperature (°C)	Recovery frequency (%)	Larvae of *G. mellonella* affected by EPNs (%)	Larvae of *T. molitor* affected by EPNs (%)
Natural grassland (NG)	02°58′43.2″ N 076°09′36.9″ W	2,963	13.9	0.0	0.0	0.0
Pasture bordering annual crops (PBAC)	02°56′57,3″ N 076°16′21,5″ W	1,757	20.0	0.0	0.0	0.0
Natural forest (NF)	02°56′0.99″ N 76°17′19.8″ W	1,863	16.4	50.0	21.7	1.7
First-growth bamboo (FGAB)	03°00′49.5″ N 076°12′41,3″ W	1,894	18.0	0.0	0.0	0.0
Coffee cultivation (CC)	02°56′0.45″ N 76°17′19.1″ W	1,852	16.6	8.3	3.3	1.7
Horticultural cultivation (HC)	03°00′49.5″ N 076°12′41,3″ W	1,894	18.0	0.0	0.0	0.0
Strawberry cultivation (SC)	02°58′43.2″ N 076°09′36.9″ W	2,963	13.9	0.0	0.0	0.0
Combined lulo and papaya cultivation (CLPC)	02°56′0,39″ N 076°17′15″ W	1,820	18.5	0.0	0.0	0.0

In total, 1,200 larvae of each host insect were exposed to all soil samples, but only 2.5% of *G. mellonella* and 0.33% of *T. molitor* larvae were affected by EPNs. Brown and flaccid larvae affected by nematodes exhibited the typical signs of Steinernematidae infection. On the other hand, the first IJs were recovered eight days after White traps settlement.

Table 2 shows EPNs isolation data in relation to types of vegetation cover and host insects. Nematodes were recovered only at sites whose soil temperatures were close to 16.5°C. Also, in both sites where EPNs were found, more *G. mellonella* larvae than *T. molitor* larvae were found dead with symptoms indicating EPNs infection. The greatest recovery frequency of EPNs was obtained from the natural forest plant cover site.

**Figure 1:**
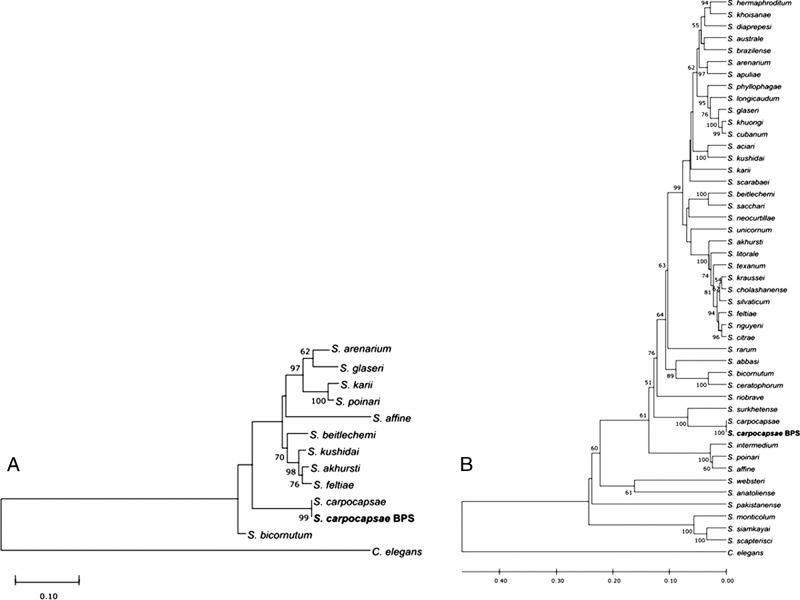
Phylogenetic tree of *S. carpocapsae* BPS. A. Phylogenetic relationships from the 18S marker. B. Phylogenetic relationships from concatenated ITS and 28S markers. The relationships were inferred by maximum likelihood estimation for the 18S marker while UPGMA was used for the concatenated markers. *Caenorhabditis. elegans* was used as an outgroup. The numbers on the nodes indicate the bootstrap values (1,000 replications).

After initial BLAST identification of the 18S, ITS, and 28S markers from the isolate recovered, 100% identity and coverage were obtained with *S. carpocapsae*. This is consistent with the phylogenetic analysis of the concatenated 18S and ITS-28S markers (Fig. 1A, B) and confirms the specific identity of the isolate named *S. carpocapsae* BPS.

### Susceptibility of *Epitrix cucumeris* and *Pandeleteius cinereus* to EPNs

**Figure 2:**
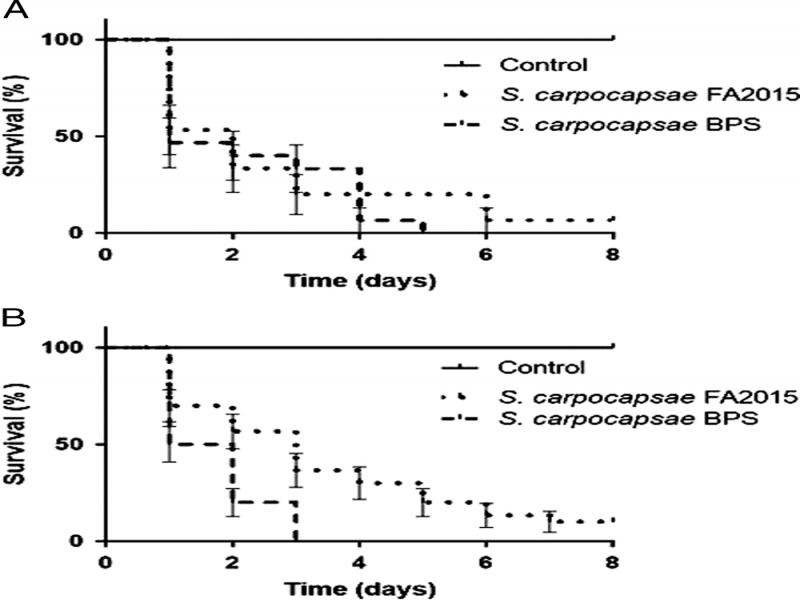
Susceptibility of two Hass avocado (*P. americana*) pest insects to *S. carpocapsae* BPS and *S. carpocapsae* FA2015. A. *E. cucumeris.* B. *P. cinereus*.

Between 0 and 6.7% of *E. cucumeris* adults survived exposure to *S. carpocapsae* (BPS and FA2015, respectively), while this result was between 0 and 10% for *P. cinereus* adults, on the other hand, 100% of both pests survived to the sterile distilled water control. This indicates susceptibility of both pests to the EPNs evaluated (Fig. 2). Analysis using the area under the curve found significant differences only in the survival of *E. cucumeris* when exposed to the two isolates (df = 2; *F* = 89.32; *P* = <0.0001). Exposure to *S. carpocapsae* BPS resulted in less survival for this pest than did the reference isolate *S. carpocapsae* FA2015, which represents a higher mortality in less time.

Adults and IJs of both *S. carpocapsae* isolates were present in 100% of the dead insects of both pests. In total, 37.1% of the nematodes found in dead *E. cucumeris* infected with *S. carpocapsae* BPS were adults, 41.1% were adults in individuals of this pest infected with *S. carpocapsae* FA2015, and the rest were IJs in both cases. In contrast, 49.8% of the nematodes found in dead *P. cinereus* insects infected with *S. carpocapsae* BPS were adults, 41.1% were adults in insects of this pest infected with *S. carpocapsae* FA2015, and the rest were IJs in both cases.

### Isolation and confirmation of bacterial symbiont

**Figure 3:**
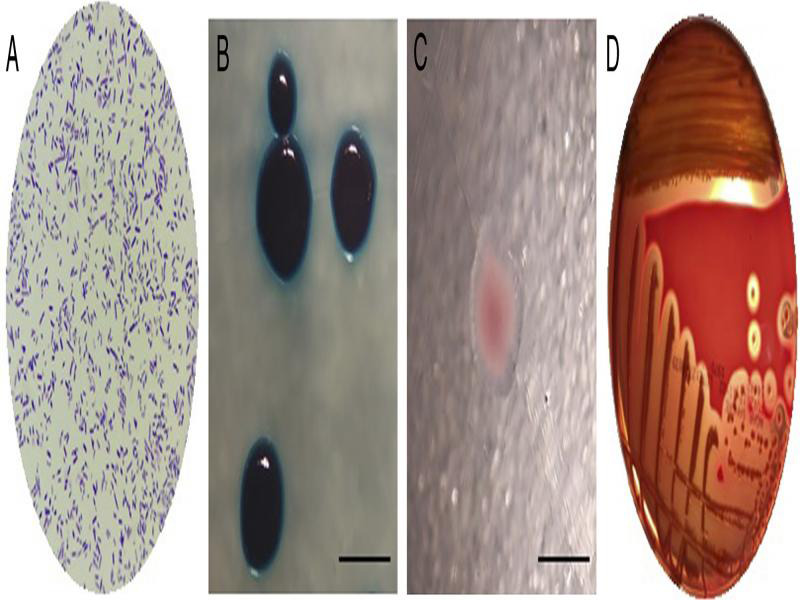
Microscopic and macroscopic morphological characteristics of the bacterial symbiont, *Xenorhabdus nematophila* BPS. A. Gram stain, 100X. B. Colonies on NBTA agar. C. Colony on MacConkey agar. D. Isolate of bacterial symbiont on blood agar, view from the back of the box. The bar measures 0.5 cm.

The bacterial symbionts of *S. carpocapsae* BPS were Gram-negative bacilli of various sizes with irregular borders and with inclusion proteins that varied in quantity and size (Fig. 3A). Macroscopically, they grew in bright, granular, convex colonies with slightly irregular borders. They were blue on NBTA agar (Fig. 3B), pink with translucent margins on MacConkey agar (Fig. 3C), and yellowish-green with total hydrolysis of red blood cells (α-hemolysis) on blood agar (Fig. 3D). In addition, *G. mellonella* larvae infected with bacterial isolate reached 100% mortality at 48 hr as evidenced by their brown color and sagging.

#### Biochemical characterization

As observed in the API biochemical gallery and under incubation conditions, the bacterial symbiont was negative for β-galactosidase, arginine-dihydrolase, lysine decarboxylase, ornithine decarboxylase, citrate assimilation, H2S production, urease, tryptophan deaminase, production of indole, acetoin production (Voges – Proskauer test), gelatinase, production of acid from glucose, mannitol, inositol, sorbitol, rhamnose, sucrose, melibiose, amygdalin, and arabinose.

#### Molecular identification

**Figure 4:**
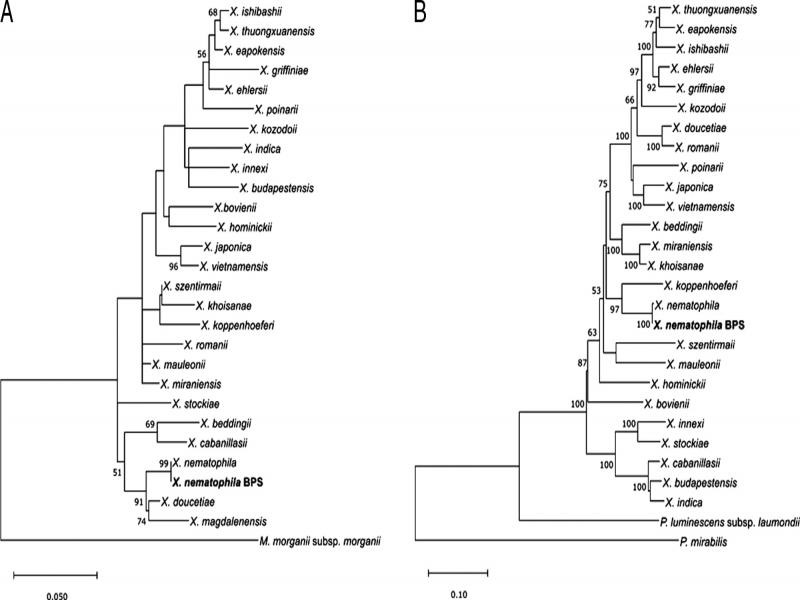
Phylogenetic tree of the bacterial symbiont *Xenorhabdus nematophila* BPS. A. Phylogenetic relationships from the 16S marker. B. Phylogenetic relationships from concatenation of *dnaN*, *recA*, *gltX*, and *rplB* markers. The relationships were inferred by maximum likelihood estimation. *Morganella morganii* subsp. *morganii* was used as an outgroup for 16S, while *Proteus mirabilis* and *Photorhabdus luminescens* subsp. *laumondii* were used for concatenated markers. The numbers on the nodes indicate the bootstrap values (≥ 50%) (100 replicates).

The initial identification, using EzBioCloud for the 16S marker and BLAST searches of the nr database of the NCBI for other markers, showed greatest similarity to the respective *Xenorhabdus nematophila* markers: 99.47% identity and 63.9% coverage were obtained for the 16S marker; 99% identity and 100% coverage were obtained for *dnaN*; 100% identity and coverage were obtained for *recA*; 99% identity and 100% coverage were obtained for *gltX*; and 100% identity and coverage were obtained for *rplB*. The phylogenetic analysis showed that the bacterial isolate was grouped at the same level with *X. nematophila* for the 16S marker as well as for the concatenated markers, thus confirming its taxonomic identity (Fig. 4A, B).

## Discussion

The recovery frequency gives an estimated of EPNs distribution between sampling points. This study found a low or uneven distribution of EPNs in the rural region evaluated in the municipality of Toribío in the department of Cauca, Colombia, which was evidenced in a low recovery frequency. Despite differences in habitats sampled and methodologies used for soil sampling and EPNs isolation, the recovery frequency obtained in this study was similar to that reported from soils of other Latin American countries such as México (6.6%, 4 positive samples out of 60) (Delgado-Gamboa et al., 2015) and Chile (7%, 97 positive samples out of 1,382) (Edgington et al., 2010), but different from others like Brazil (23.2%, 73 positive samples out of 315) (Foelkel et al., 2017).

In contrast, in this study only one isolate of *S. carpocapsae* was recovered and multiplied from soils of both natural habitats (natural forest) and crops (coffee cultivation) between 1,757 and 2,963 m.a.s.l, while in México Delgado-Gamboa et al. (2015) recovered four EPN isolates (three *S. carpocapsae* and one *S. websteri*) from soils of agricultural areas planted to agave (*Agave angustifolia* Haw) between 1,400 and 1,600 m.a.s.l, in Chile Edgington et al. (2010) recovered 101 EPN isolates, 94 *Steinernema* sp. (*S. australe, S. unicornum*, and *S. feltiae*) and 7 *Heterorhabditis* sp.( *H.* cf*. safricana*), from soils of natural habitats and agricultural areas between 0 and 2,499 m.a.s.l, while in Brazil Foelkel et al. (2017) recovered five EPN isolates which were closely related to *Oscheius* sp. from soil samples of an apple orchard (cultivar Eva) at 865 m.a.s.l., which points the variable and cosmopolitan distribution of EPNs (Rizvi et al., 2012). However, in this study both *G. mellonella* and *T. molitor* were used in combination as baits for EPNs recovery, in México and Chile studies only last-instar larvae of *G. mellonella* were used, while in Brazil *T. molitor* and *Anastrepha fraterculus* (Diptera: Tephritidae) were used independently as baits for EPNs recovery.

Regarding studies in Colombia, in terms of recovery frequency our result is similar to that reported by López-Núñez et al. (2007) who, in departments of Caldas, Quindío, Risaralda and Cundinamarca, found 3% of samples positive for EPNs (28 out of 945) corresponding to 26 isolates of *Steinernema* sp. (*S. websteri* and four undescribed *Steinernema* sp. taxa) and two of *Heterorhabditis* sp. (both undescribed) from soils of coffee crops and natural habitats between 1,203 and 1,478 m.a.s.l using only last-instar *G. mellonella* larvae for EPNs recovery. However, analyzing only positive sites for EPNs, López-Núñez et al. (2007) obtained 2 and 6% of recovery frequency in forests, which is lower than that obtained in this study for natural forest (50%), and indicates a higher distribution of EPNs between sampling points of this habitat in this study. In contrast, they found recovery frequencies for EPNs from 4 to 18% for areas cultivated in coffee (*C. arabica*), which was similar to the results obtained in this study for coffee cultivation (8.3%).

The differences between the recovery frequencies found in this study compared to the results of the other studies referenced from Latin America and Colombia could be due to that factors such as soil type, distribution of suitable hosts, physiological and behavioral adaptations are key factors affecting the distribution of EPN species (Adams et al., 2006; Stuart et al., 2006). However, some of these resources have heterogeneous distribution, therefore, nematode populations are highly aggregated (Ettema and Lal, 2006).

Despite EPNs were isolated at two kind of habitats; one cultivated site (CC) and at one natural habitat (NF), the greater recovery frequency of the second one indicates that, despite the fact that natural habitats contain greater diversity of insects controlled by one or another natural enemy, it is likely that at NF there is an ecological imbalance which favors the incidence of EPNs in this habitat where they were widely distributed in the soil (Campos-Herrera et al., 2007; Jaffuel et al., 2018). By the other hand, while monocultures may have greater availability of hosts susceptible to attack by EPNs, the continuous use of pesticides can limit availability and negatively affect presence of biocontrol agents, which could be the reason for a low recovery frequency of EPNs at CC or even the no recovery of EPNs in other crop sites evaluated in this study.

In terms of abundance, which gives an estimated about the distribution of EPNs between sampling sites, in this study the EPNs abundance, was lower than registered by López-Llano and Soto-Giraldo (2016) whom obtained 88.2% of abundance (15 positive sites for EPNs out of 17) from sugarcane crops (*Saccharum officinarum*) in the department of Caldas, in which were recovered 15 isolates of *Steinernema* sp. and 6 of *Heterorhabditis* sp, while Melo et al. (2009) obtained 74% of abundance (17 positive sites for EPNs out of 23) from crops between 990 and 1,660 m.a.s.l. in departments of Quindío, Risaralda, Caldas and Cauca. However, in contrast for what was done in this study, both other ones mentioned above used only last-instar larvae of *G. mellonella* for EPNs recovery.

Although we allegedly detect EPNs at two sites, it was only possible to multiply and maintain the isolate from NF. This may have been due to ignorance of biological, ecological, and temperature conditions required for these nematodes to infect susceptible hosts and indicate a need for additional studies in the area using other detection/extraction methods and/or host insects.

Even though EPNs were allegedly detected at both sites through using the two host insects evaluated, it was evident that *G. mellonella* was generally more efficient at detecting EPNs than was *T. molitor*. This indicates that *G. mellonella* is more susceptible to the EPNs potentially present at both sites. Nevertheless, there were more *G. mellonella* larvae affected by EPNs at the NF site than at the CC site, while the same number of affected *T. molitor* larvae was obtained at both sites. This can be attributed to the abundance and nature of the EPNs potentially present at the CC site.

EPNs of the genera *Heterorhabditis* and *Steinernema* have been found on all continents: in South America they have been recovered in eight countries (Stock, 2015; Stuart et al., 2015). In Colombia, *Steinernema* species reported include *S. websteri* (López-Núñez et al., 2007), a new species named *S. colombiense* (López-Núñez et al., 2008), and *S. kraussei* (Melo et al., 2009). The isolate obtained in this study was identified as *S. carpocapsae*, a nematode that in South America had previously only been reported in Argentina, Brazil, and Perú (Cabi.org, 2018; San-Blas et al., 2019).

*Steinernema carpocapsae* is found throughout the world (Hominick, 2002), but this species is not frequently isolated (Yan et al., 2016). This is probably due to the species’ ambush behavior. When *G. mellonella* is used as a target insect to isolate *S. carpocapsae*, its field frequency is underestimated (Mráček et al., 2005). Despite this, *S. carpocapsae* has been recovered in grassland areas (602-677 m.a.s.l, average soil temperature of 11°C) (Campos-Herrera et al., 2007) and in forests (1,700 m.a.s.l, soil temperature of 19°C) (Yan et al., 2016). These factors are similar to those in the areas sampled in this study.

Although it has been reported that *S. carpocapsae* can infect about 95% of the larvae of *T. molitor* (San-Blas et al., 2012), this study found that a greater number of *G. mellonella* larvae were affected. This could be attributable to the poor mobility of *T. molitor* in soil combined with the ambush behavior of *S. carpocapsae*. This EPN attacks high-mobile insects more efficiently (Banu et al., 2017). Our findings are consistent with those obtained in a study by Yan et al. (2016) in which *S. carpocapsae* isolate was exposed to the same two insect larvae, but nematodes were only recovered from *G. mellonella*.

*Steinernema carpocap*sae has been shown to establish a specific and exclusive symbiosis with *X. nematophila* (Cowles and Goodrich-Blair, 2008) as its only bacterial symbiont, so its identification as the bacterial isolate of this study was expected. The isolate showed morphological characteristics typical of *X. nematophila* in phase 1 (Akhurst and Boemare, 2015), while most of its biochemical characteristics obtained. Our results agree with what has been reported for this species elsewhere, except for our negative results for assimilation of citrate, gelatinase activity and acid production from glucose. These differences may be attributable to the fact that metabolic specialization can be found even within the same species (Ponomarova and Patil, 2015) caused by factors such as temperature that affect enzymatic activity (Peterson et al., 2007). Therefore, the conditions in which the biochemical tests were carried out can give rise to different results to what is reported for bacteria of the same species.

*Steinernema carpocapsae* is the EPN species most commonly used to control insect pests associated with the soil surface and leaf area (Lacey and Georgis, 2012). Although its potential as a control agent has been demonstrated in other insects of the Curculionidae family such as the red weevil, *Rhynchophorus ferrugineus* (Manachini et al., 2013) and the coffee borer beetle, *Hypothenemus hampei* (Manton et al., 2012), there had been no previous reports of its efficacy for controlling *P. cinereus*. Therefore, this is the first report of its susceptibility to *S. carpocapsae*. On the other hand, the efficacy of *S. carpocapsae* for controlling two members of the Chrysomelidae family, western corn rootworms, *Diabrotica virgifera* (Journey and Ostlie, 2000), and potato flea beetles, *Epitrix* spp. (Parker et al., 2012), has already been demonstrated. This study has shown that *P. cinereus* and *E. cucumeris* adults which damage Hass avocado crops are susceptible to both isolates of *S. carpocapsae*, and that S*. carpocapsae* BPS has greater capacity to cause less survival of *E. cucumeris* adults.

The results obtained for susceptibility of both pests to *S. carpocapsae* are important because these pests affect Hass avocado crops (*P. americana*) in Colombia. They eat the leaves of the plants causing cuts that decrease leaf area thereby reducing the rate of photosynthesis which in turn reduces the yield of the plant. Similarly, *P. cinereus* causes damage to the ovary, petals, and newly formed fruit causing them to fall from the trees (Londoño et al., 2014). Consequently, our findings of in vitro susceptibility of both pests to *S. carpocaosae* now need to be corroborated by field studies that can determine the potential of these EPNs for controlling these two pests of economic interest to Hass avocado cultivation (*P. americana*) in Colombia.

## Conclusions

These results are the first record of *S. carpocapase* and its bacterial symbiont in Cauca, Colombia. They establish its potential for controlling two pests that affect Hass avocado crops (adults of *P. cinereus* and *E. cucumeris*). Additional studies are needed to complement and confirm these results, especially field trials to determine the possibility of using these nematodes in integrated pest management programs for these pests.
